# Relationship Between Intermuscular Synchronization of Upper Leg Muscles and Training Level in Karate Kumite Practitioners

**DOI:** 10.3390/jfmk10030234

**Published:** 2025-06-20

**Authors:** Velimir Jeknić, Milivoj Dopsaj, Nenad Koropanovski

**Affiliations:** 1 Faculty of Sport and Physical Education, University of Belgrade, 11030 Belgrade, Serbia; milivoj.dopsaj@fsfv.bg.ac.rs; 2 Department of Criminalistics, University of Criminal Investigation and Police Studies, 11080 Belgrade, Serbia

**Keywords:** muscular adaptations, thigh muscles, combat sports, coordination indexes, tensiomyography

## Abstract

**Objectives**: This study aimed to compare the involuntary stimulated neuromuscular response of thigh muscles in karate subgroups and non-athletes. We investigated whether karate training creates neuromuscular adaptations and if the synchronization of knee flexor and extensor muscles in karate practitioners is level-dependent. **Methods**: The study included 7 elite karate athletes (KE), 14 sub-elite karate athletes (KSE), 16 individuals with basic karate training (KB), and 14 non–athletes (NA). Tensiomyographic (TMG) measurements were obtained from the rectus femoris, vastus medialis, vastus lateralis, biceps femoris, and semitendinosus muscles. Indexes of Intermuscular Synchronization (IIS) were calculated for contraction time (Tc), total contraction time (TcT), and rate of muscle tension development (RMTD) as variables for the observed muscles of a given muscle group (extensors of the dominant leg, flexors of the dominant leg, extensors of the non-dominant leg, and flexors of the non-dominant leg). **Results**: Statistically significant differences were observed in the intermuscular synchronization indexes between karate experience levels and non-athletes. Compared to non-athletes, elite (KE), sub-elite (KSE), and beginner karateka (KB) all demonstrated shorter contraction time indexes in dominant knee extensors (*p* = 0.042, 0.040, and 0.013, respectively). In the non-dominant flexors, KE exhibited significantly better synchronization than KSE (*p* = 0.001), KB (*p* = 0.033), and NA (*p* = 0.002). For the total contraction time index, both KSE and KB outperformed NA in dominant extensors (*p* = 0.023 and *p* = 0.008), while KE showed superiority in non-dominant extensors and flexors compared to all other groups (*p*-values ranging from 0.002 to 0.038). Significant RMTD differences were found in the dominant leg between KE and KSE (*p* = 0.036) and KE and KB (*p* = 0.001), as well as in the non-dominant leg between KE and KB (*p* = 0.011) and KE and NA (*p* = 0.025). These findings were accompanied by statistical powers exceeding 0.80 in most cases, underscoring the robustness of the observed differences. **Conclusions**: These findings highlight that muscle coordination patterns, as revealed through non-invasive TMG-based indexes, are sensitive to training level and laterality in karate practitioners. Importantly, elite athletes demonstrated more synchronized activation in key muscle groups, suggesting a neuromuscular adaptation specific to high-level combat sports. From a biomechanical perspective, improved intermuscular synchronization may reflect optimized neural strategies for stability, speed, and efficiency—key components in competitive karate. Thus, this method holds promise not only for performance diagnostics but also for refining individualized training strategies in combat sports and broader athletic contexts.

## 1. Introduction

Human movements, particularly those of elite athletes, reveal the substantial precision and coordination of the neuromuscular system [1]. Regarding neural adaptations in intermuscular coordination, three mechanisms are said to promote gains in strength performance: increased activation of agonists, decreased activation of antagonists, and activation of synergists [2]. The coordinated activation of multiple muscles during rhythmic movements is commonly referred to as a muscle synergy [3]. Motor training may encourage the development of new muscle synergies for new tasks, change the structure of existing muscle synergies, or change the manner in which existing muscle synergies are recruited [4]. In sports activities, almost every action is performed with the concomitant contribution of multiple joint motion muscle synergies. It is therefore not surprising that a continuous relative phase approach has been widely investigated in running [5], cycling [6], rowing [7], gymnastics [8], breaststroke swimming [9], etc. Contrary to the use of phase relationships as a measure of coordination, previous studies have also demonstrated that functionally and anatomically related muscles show motor unit (MU) synchronization [10,11].

Karate is a specialized martial art that demands nerve and muscle coordination [12]. Under strict rules, athletes need to control the velocity of the attack so that it attains the highest possible velocity during the course of the punch, but reaches the target with a velocity close to zero [13]. Such a maneuver can be easily perceived by the opponent if the stroke is not fast enough and if the time required for the braking process is not short enough [14]. Achieving a high level of performance in a complex structured sport such as karate is determined by the physical preparation, technique, fighting tactics, and mental state of the competitor [15]. In particular, the competitor must react as quickly as possible to visual or acoustic stimuli with maximum speed, power, and control of movement performance [16]. In addition to the control of static positions, there is also the requirement of controlling dynamic movements, because the techniques that are used are performed in an extremely dynamic, precise, and specifically coordinated manner [15,17]. In other words, each technique used in karate is a complex motor skill that requires neuromuscular control, motor prediction, and highly developed proprioception [18], and therefore represents a very demanding activity from a coordinative viewpoint [19]. Although previous research on muscular coordination in karate was mainly focused on the activation of muscles during punches and kicks [20,21,22,23], there is a growing interest in different approaches to the examination of muscle coordination strategies in human locomotion [24,25]. Namely, the findings of Garcia-Retortillo et al. [24] indicate the existence of the general physiological principles of intermuscular coordination for different fundamental movement patterns, so that muscle network interactions do not depend on the specific movement, but rather reflect the role that pairs of muscles play during each movement.

Regarding specific karate adaptations, the neuromechanical characteristics of functionally and anatomically related muscle synergies remain relatively unexplored. Coordinative patterns in karate have mostly been investigated by analyzing limb kinematics [26,27]. Khanzadeh et al. [28] investigated the muscle stimulation time patterns of the leg, hip, and trunk muscles using electromyography during each of the implementation phases of the Ura Mawashi Geri technique separately. The authors concluded that the stability muscles in joints are involved in the action earlier than the dynamic muscles at their activity phase. Quinzi et al. [29] analyzed the phase relationships between hip and knee flexion and extension in elite karateka using a stereophotogrammetric system. They compared the coordinative pattern of no impact and impact kicking actions in order to investigate differences between the intralimb coordination adopted in the two kicks. The authors highlighted a delayed flexion of the hip with respect to the knee during the central portion of the movement in both kicks, but different patterns in the terminal portion, depending on impact. Likewise, Błaszczyszyn et al. [12] examined the effectiveness of the Mae-Geri technique and concluded that it requires a dynamic sequence of movements involving the trunk, pelvis, knee, ankle, and foot, resulting in the activation of neuromuscular support in the lower limb and enabling synergy from the pelvis to the foot. Given the contents and nature of karate training, which have been shown to improve physical fitness and nerve and muscle coordination [30,31,32], it is plausible that this form of training may enhance neuromuscular coordination and increase lower limb muscle activity in a sport-specific manner. The quantification of muscle coordination has usually been studied through the decompensation of surface EMG recordings into muscle synergies [2]. Still, it remains unclear whether it is possible to quantify intralimb muscle coordination from a memorized, involuntary perspective in isolated muscle groups (knee flexors and extensors), without the presence of crosstalk in the observed muscle signals and without the influence of the athlete’s motivation.

The goal of the present study is to investigate the effects of training expertise on intermuscular synergy coordination in adult male karate practitioners. According to the previously presented evidence, we hypothesize that the Index of Intermuscular Synchronization (IIS) profiles will differ based on the level of training and competitive success. Furthermore, we speculate that neuromuscular activity must be highly coordinated in elite karate athletes. In order to test this hypothesis, we examined individual synergistic muscles sharing anatomical and functional features (knee flexors and extensors) via the tensiomyography (TMG) method and the introduced IIS, which is a completely innovative analytical approach when it comes to measuring the contractile properties of skeletal muscles with this technique.

## 2. Materials and Methods

### 2.1. Participants

**Figure 1:**
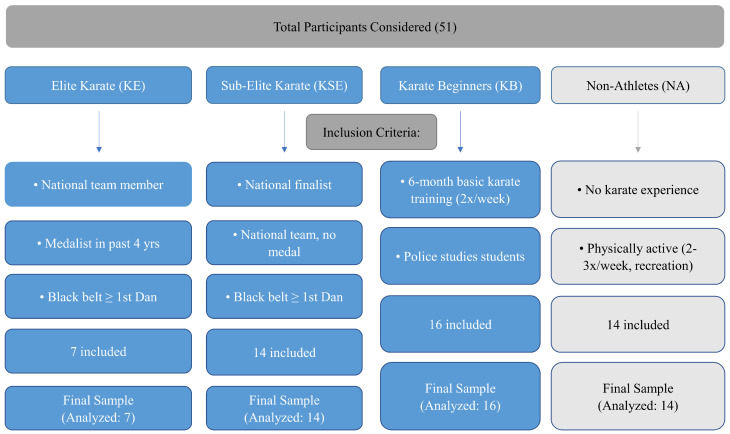
Inclusion Criteria for Participants.

The research included a sample of 7 elite male karate competitors (KE), 14 sub-elite male karate competitors (KSE), 16 male individuals that have undergone basic karate training (KB), and 14 physically active and healthy controls of the same age and gender as the karate subgroups—non-athletes (NA)—Table 1. The inclusion criteria for the above-mentioned groups are given at the Figure 1.

In the KE, KSE, and KB groups, the dominant leg (D) was defined as the leg used to perform the movement, while the non-dominant leg (ND) was defined by its stabilizing or supporting role [33]. In particular, the dominant leg is defined as the pushing leg in the fighting stance (responsible for the largest share of horizontal movement production) and at the same time the leg that is said to be used most frequently in kicking techniques for scoring points. The non-dominant leg is in the front position and stops the forward movement of the body, provides balance during the kick and controls the distance between the karateka and his opponent. The dominant leg in the NA group was determined by filling out a questionnaire for assessing the dominant lateralization of the lower extremities [34].

None of the participants reported any medical problems or recent injuries that could compromise the tested performance. All participants were fully informed about the potential risks associated with the research and signed informed consent forms previously approved by the Research Ethics Committee of the Faculty of Sports and Physical Education of the University of Belgrade (No. 484-2), in accordance with the criteria of the Declaration of Helsinki for research involving human beings.

### 2.2. Procedures

Subjects were tested in the morning hours and agreed not to perform any exhaustive physical activity to allow for a recovery time of 48 h. They also refrained from consuming products containing stimulants or tranquilizers on the day of the test. The examination was conducted by a team of researchers consisting of two persons experienced in testing with the TMG apparatus. The experiment was conducted within one test. It consisted of anthropometric measurements followed by TMG assessment of neuromuscular performance. Body height (BH) was measured with an accuracy of 0.1 cm, and body mass (BM) with an accuracy of 100 g. After that, body mass index (BMI = BM/BH2) was calculated. The involuntary contractile characteristics of the muscles of the lower extremities—the rectus femoris, vastus medialis, vastus lateralis, biceps femoris, and semitendinosus—were recorded by measuring the response of these muscles to an induced electrical stimulus (provoked with two self-adhesive electrodes),using TMG equipment (TMG100, System electrostimulator, TMG-BMC d.o.o., Ljubljana, Slovenia), on the lower right and left extremity, more precisely the thigh muscles.

One muscle was tested on the right and left leg, after which the focus moved to the next muscle. The rectus femoris, vastus medialis, and vastus lateralis were tested while the subject was in the supine position (lying on their back), while the biceps femoris and semitendinosus were tested while the subject was in the prone position (lying on their stomach). The knee joint was fixed at an angle of 120° [35]. The measurement site was carefully determined (visually and by palpating) as the point of maximum movement of the muscle belly during voluntary contraction [36]. A sensor (GK40, Panoptik, Ljubljana, Slovenia) that detects changes in the belly of the muscle caused by electrical stimulation was placed between the electrodes to obtain data on the involuntary contractile properties of the muscle [35]. All measurements were performed by the same research team using six electrical stimuli lasting 1 ms (10, 25, 50, 75, 100, and 110 mA). Ten seconds of recovery were allowed between measurements to minimize the effects of potentiation and fatigue. For each participant, only the signal that recorded the highest Dm (maximum radial displacement of the muscle belly) was considered for analysis [37].

The following contractile characteristics were measured and subsequently derived: Dm—maximum vertical movement of the muscle; (mm)—maximum movement of the belly of the muscle caused by electrostimulation; Td—delayed contraction time (ms), the time required to reach 10% of the maximum displacement of the muscle belly; Tc—contraction time (ms), the time required to reach 10–90% of the maximum vertical movement of the belly of the muscle; TcT—total contraction time (mm), a derived variable, obtained by summing the contraction time and delayed contraction time (Tc + Td); and RMTD—rate of muscle tension development, i.e., the speed of vertical muscle movement as an analogy of contraction speed (mm/ms), a derived variable, calculated as the ratio of maximum vertical muscle movement to contraction time (Dm/Tc) [38]. For each of the selected muscles, these five variables were calculated to assess the involuntary neuromuscular contractile characteristics.

### 2.3. TMG Indexes of Intermuscular Synchronization (IIS)

Indexes of Intermuscular Synchronization were defined as follows:

For each individual variable of the TMG that was examined (Tc, TcT, and RMTD), the degree of variability was determined in relation to the tested extensor muscles and the tested flexor muscles of the knee joint. In this way, the variability of the measured functional characteristics of muscle contraction was a measure of the synchronization of different muscles within one functional group (for knee flexors, the vastus lateralis, vastus medialis, and rectus femoris; for knee flexors, the biceps femoris and semitendinosus) of both dominant and non-dominant legs. In other words, the more similar the time Tc in the vastus lateralis, vastus medialis, and rectus femoris was, that is, the less it varied, the more synchronized the given functional muscle group was. In this way, the following variables were used in the work:

- For the dominant leg:
  1. IIS_AVG_Tc_Q_D, which is calculated in the following way:
    (RF_Tc + VL_Tc + VM_Tc)/3, expressed in ms.
  2. IIS_cV_Tc_Q_D, which was calculated as a coefficient of variation and represents the quotient between the standard deviation and the average value of Tc for RF, VL, and VM of the dominant leg, expressed as a percentage.
  3. IIS_AVG_Tc_H_D, which is calculated in the following way:
    (BF_Tc + ST_Tc)/2, expressed in ms.
  4. IIS_cV_Tc_H_D, which is calculated as a coefficient of variation and represents the quotient between the standard deviation and the average value of Tc for the biceps femoris and semitendinosus of the dominant leg, expressed as a percentage.
- For the non-dominant leg:
  1. IIS_AVG_Tc_Q_ND, which is calculated in the following way:
    (RF_Tc + VL_Tc + VM_Tc)/3, expressed in ms.
  2. IIS_cV_Tc_Q_ND, which was calculated as a coefficient of variation and represents the quotient between the standard deviation and the average value of Tc for the RF, VL, and VM of the non-dominant leg, expressed as a percentage.
  3. IIS_AVG_Tc_H_ND, which is calculated in the following way:
    (BF_Tc + ST_Tc)/2, expressed in ms.
  4. IIS_cV_Tc_H_ND, which was calculated as a coefficient of variation and represents the quotient between the standard deviation and the average value of Tc for the biceps femoris and semitendinosus of the non-dominant leg, expressed as a percentage.

The IIS was obtained for the remaining two TMG variables with the same calculation procedures:

TcT (IIS_AVG_TcT_Q_D, IIS_cV_TcT_Q_D, IIS_AVG_TcT_H_D, IIS_cV_TcT_H_D, IIS_AVG_TcT_Q_ND, IIS_cV_TcT_Q_ND, IIS_AVG_TcT_H_ND, IIS_cV_TcT_H_ND).

And RMTD (IIS_AVG_RMTD_Q_D, IIS_cV_RMTD_Q_D, IIS_AVG_RMTD_H_D, IIS_cV_RMTD_H_D, IIS_AVG_RMTD_Q_ND, IIS_cV_RMTD_Q_ND, IIS_AVG_RMTD_H_ND, IIS_cV_ RMTD_H_ND).

In this way, the area of intermuscular synchronization in the knee joint of the dominant and non-dominant leg was described with 24 variables in four groups of subjects that differed in their level of karate skill, namely elite karate athletes, sub-elite karate athletes, a basic training group, and active healthy controls.

### 2.4. Statistical Analysis

Descriptive analysis was applied to describe the quantitative measures of the studied variables, where the mean value of the variables (Mean), standard deviation (SD), minimum (Min), maximum (Max), and coefficient of variation (cV%) were calculated. The normality of the data distribution was assessed using the Shapiro–Wilk test for each muscle group and participant category. A univariate one-way analysis of variance (ANOVA) was used to determine differences between the groups, according to the lateral dominance, and the *T*-test was used to determine the differences between pairs of variables. The effect of the difference between the groups was determined by application of the Bonferroni correction, where the value of Eta^2^ was calculated, as well as the power of the analysis.

All statistical analyses were performed using the SPSS 20.0 software package (IBM, Chicago, IL, USA), while the 95% probability limit for the value *p* < 0.05 was used for the level of statistical significance [39].

## 3. Results

The IIS of the extensor and flexor muscles in the knee joint in the dominant leg for the contraction time (Tc) parameter showed statistically significant differences between the groups for the variables IIS_AVG_Tc_Q_D and IIS_cV_Tc_Q_D. Regarding the IIS_AVG_Tc_Q_D, all three karate subgroups had a shorter average contraction time compared to the NA group (Table 2). More precisely, KE contraction time was significantly shorter than NA (*p* = 0.042), KSE than NA (*p* = 0.040), and also KB was significantly shorter than NA (*p* = 0.013). In addition, for the variable IIS_cV_Tc_Q_D, KE had significantly lower cV than NA (*p* = 0.042). Also, it is necessary to emphasize the observed clear nominal linear tendency of IIS_cV_Tc_Q_D reduction as the level of training, i.e., sports success, increases (Table 2).

The results of the IIS of extensor and flexor muscles in the knee joint of the non-dominant leg for the Tc parameter show statistically significant differences between the groups for the variables IIS_AVG_Tc_Q_ND and IIS_AVG_Tc_H_ND (Table 3). At the individual level, KSE had significantly shorter contraction times when compared to NA (*p* = 0.041) within the variable IIS_AVG_Tc_Q_ND. Regarding the variable IIS_AVG_Tc_H_ND, KE had significantly shorter times than KSE, KB, and NA (*p* = 0.001, *p* = 0.033, and *p* = 0.002, respectively).

The results of the IIS of the dominant extensor and flexor muscles in the knee joint for the total contraction time (TcT) parameter show a statistically significant difference between the groups for the variable IIS_AVG_TcT_Q_D (Table 4). Specifically, the KSE and KB groups had significantly lower times than the NA group (*p* = 0.023 and *p* = 0.008, respectively).

The results of the IIS of the extensor and flexor muscles in the knee joint of the non-dominant leg for the TcT parameter show statistically significant differences between the groups for the variables IIS_AVG_TcT_Q_ND and IIS_AVG_TcT_H_ND (Table 5). Specifically, regarding the IIS_AVG_TcT_Q_ND variable, all three karate subgroups (KE, KSE, and KB) had significantly shorter times than the NA group (*p* = 0.022, *p* = 0.008, and *p* = 0.017, respectively). Regarding the IIS_AVG_TcT_H_ND variable, KE had significantly shorter TcT than KSE, KB, and NA groups (*p* = 0.002, *p* = 0.038, and *p* = 0.002, respectively).

The results of the IIS of the extensor and flexor muscles in the knee joint of the dominant leg for the rate of muscle tension development (RMTD) parameter indicate statistically significant differences between the groups for the IIS_cV_RMTD_Q_D and IIS_AVG_RMTD_H_D variables (Table 6). In the analysis of the IIS_cV_RMTD_Q_D variable, variation in the KE was significantly lower than in KSE (*p* = 0.036) and KB (*p* = 0.001). Also, an analysis of the IIS_AVG_RMTD_H_D variable showed that the KE and KSE results were significantly higher than NA (*p* = 0.014 and *p* = 0.006, respectively).

The results of the IIS of the extensor and flexor muscles in the knee joint of the non-dominant leg for the RMTD parameter show a statistically significant difference between the groups for the IIS_AVG_RMTD_Q_ND and IIS_AVG_RMTD_H_ND variables (Table 7). Specifically, within the variable IIS_AVG_RMTD_Q_ND, KE was significantly higher than for the KB and NA groups (*p* = 0.011 and *p* = 0.025, respectively). The variable IIS_AVG_RMTD_H_ND was significantly higher in the KE than in the NA group (*p* = 0.016).

## 4. Discussion

The aim of the present study was to investigate and compare the neural strategies underlying intermuscular synchronization between elite karate athletes, sub-elite karate athletes, basically trained karateka, and non-athletes using TMG technology. The results present intermuscular coordination through indexes of average values and coefficients of variation for the dominant and non-dominant flexor and extensor muscles in the knee joint (rectus femoris, vastus lateralis, vastus medialis, biceps femoris, and semitendinosus) for the Tc, TcT, and RMTD parameters. The results provide evidence in support of our hypothesis that there is significant coherence in the TMG indexes of knee flexor and extensor muscle synchronization. Furthermore, our hypothesis that the IIS has a hierarchical structure of influence that depends on the level of training and success in karate was confirmed.

While the initial hypothesis anticipated a progressive gradient in neuromuscular coordination from elite athletes to non-athletes (i.e., KE < KSE < KB < NA), the actual results revealed more complex and variable trends. In some parameters, elite karateka (KE) were distinct from all other groups (Table 3 and Table 5), while in others, sub-elite and basic practitioners did not differ significantly from non-athletes (Table 7). This variability may reflect the influence of multiple overlapping factors beyond training volume or competition level alone—such as individual neuromuscular efficiency, variability in training quality, or adaptations specific to task demands rather than general physical conditioning. It is also possible that sub-elite and basic karateka experience a plateau in neuromuscular adaptation without reaching the refined motor control evident in elite athletes. These inconsistencies highlight the need for more nuanced models that consider skill specialization, training content, and motor learning stage rather than assuming a strict linear progression across training levels.

Among the muscles recorded in this study, the rectus femoris, vastus medialis, and vastus lateralis are synergists that are on a functional level responsible for knee extension [40]. Tc and TcT IIS differences in dominant and non-dominant extensor muscles suggest that all subgroups of karate athletes are more synchronized than the NA group, supporting the view of a central drive regulating the common fine activation structure of these muscles. It should be noted here that the differences between the KB and NA that occur in IIS_AVG_TcT_Q_D and IIS_AVG_TcT_Q_ND (Table 3 and Table 4) suggest a cumulative training effect resulting from training episodes [41]. These results are consistent with the claim that athletes who regularly participate in planned physical activities develop sport-specific muscle qualities resulting from adaptation to training [42]. A similar change was found in a study by Jemili et al. [43], where changes in the form of a reduction in the quadriceps muscle contraction time of karate athletes occurred after 3 months of intensive karate training. In addition, Pozo et al. [44] concluded that a group of international karateka activated a greater number of quadriceps fast motor units during the attack phase of a Mae Geri kick compared to a national level group. RMTD differences were found to be significant between the KE and other subgroups in the IIS_cV_RMTD_Q_D and IIS_AVG_RMTD_Q_ND variables, which confirms the fact that the specificity of adaptation increases with the level of sport mastery [41]. There are several studies that support this claim. For instance, Matsunaga & Kaneoka [45] found some pronounced differences between differently leveled groups, namely during a badminton smash shot, where one particular muscle synergy was found only in the advanced players, revealing that enhanced performance in badminton smash shots may be related to neural strategies developed by training. In a study by Goethel et al. [14], the authors suggest that it is possible that one of the main effects of high-level training in karate is the development of faster movements due to the improvement and optimization of perceptual and reaction skills as well as neuromuscular control, allowing for improved decision-making and consequently faster reactions as demonstrated by the elite athletes performing the Gyaku Tsuki punch. In the aforementioned study, the elite group responded much faster than the sub-elite group in each timing parameter.

Taking into consideration the different functions of the biceps femoris and semitendinosus during rotations [46], only elite karate athletes developed a high level of synchronization of these two muscles, which is reflected in statistically significant differences in “time” variables (Tc and TcT) between the KE and all the other observed groups. Also, the KE showed a significant difference in the “speed” variable (RMTD) in comparison with the NA group. An additional specificity is that a high level of compliance, or coactivation, must exist in order for the non-dominant leg to maintain dynamic balance and stability [47] when performing kicks with the dominant leg. The biceps femoris and semitendinosus muscles are synergists that, on a functional level, are responsible for knee flexion [48]. The flexor muscles of the non-dominant leg are not exposed to antagonistic activations, as is the case with the flexors of the dominant leg [49]. Instead, they play a significant role in assisting the execution of fast external rotations during kicks, followed by internal rotations afterwards. The quick return of the body segments to their initial position by the aforementioned activation of the hamstring muscles is motivated by the need to satisfy another aspect of point karate scoring—Japanese “Zanshin”, which represents a specific posture after finishing the attacking technique [43,50]. One of the main findings of a study by Hoebling et al. [47] is that higher-ranked fighters demonstrate better balance of the supporting (non-dominant) leg, which allows for horizontal movement towards the opponent while the dominant leg is raised high for the attack. Also, in a study that compared elite and amateur karateka [32], the results suggest that elite karateka demonstrate refined control of dynamic balance, which is particularly pronounced during kicks, with one leg raised high while the karateka performs an attacking technique. The authors [32] explained this phenomenon by the longer time exposure of elite karate fighters to complex motor tasks, such as different changes in the center of gravity, body rotations, and stances on one leg, which require a high level of coordination among the synergist muscles of the non-dominant leg’s hamstring muscles.

Our approach to intermuscular synchronization effectively captured the orchestration of neuromuscular adaptations, as evident in the specific utilization of synergist muscle responses within their functional groups. Additionally, the hierarchy of neuromechanical strategies employed to activate synergists exhibited a notable correlation with both the level of training and competition success. To give an illustration, 17 statistically significant differences in the Q and H muscle IIS were found between the karate subgroups and non-athletes, as follows: Between the KB and NA groups—three (IIS_AVG_Tc_Q_D, IIS_AVG_TcT_Q_D, and IIS_AVG_TcT_Q_ND); the KSE and NA groups—five (IIS_AVG_Tc_Q_D, IIS_AVG_Tc_Q_ND, IIS_AVG_TcT_Q_D, IIS_AVG_TcT_Q_ND, and IIS_AVG_RMTD_H_D); and between the KE and NA groups—nine differences (IIS_AVG_Tc_Q_D, IIS_cV_Tc_Q_D, IIS_AVG_Tc_H_ND, IIS_AVG_TcT_Q_ND, IIS_AVG_TcT_H_ND, IIS_cV_RMTD_Q_D, IC_AVG_RMTD_H_D, IIS_AVG_RMTD_Q_ND and IC_AVG_RMTD_H_ND). Between the karate subgroups themselves, we found seven statistically significant differences. The KE group had the most IIS differences in relation to the other two karate subgroups: when compared to KB—four differences (IIS_AVG_Tc_H_ND, IIS_AVG_TcT_H_ND, IIS_cV_RMTD_Q_D and IIS_AVG_RMTD_Q_ND) and when compared to KSE—three differences (IIS_AVG_Tc_H_ND, IIS_AVG_TcT_H_ND and IIS_cV_RMTD_Q_D). The lowest variability was found in the extensor (variable Tc) and flexor (variable RMTD) muscles of the dominant (kicking) leg of the KE group (Table 2 and Table 6). This represents the high level of intermuscular synchronization in the KE group conditioned by the fast kicking requirements in the knee extension phase of the movement and strength requirements of the breaking action of the antagonist hamstring muscles, due to the specific training strategies adopted in order to better control the kicking impact imposed by the karate competition rules [51]. The results of the TMG analysis presented in this study show differentiated neuromuscular adaptation by elite karate athletes, possibly as a result of greater technical refinement. The tensiomyography method offers a novel perspective on intermuscular coordination to help better understand the complex relationships within the agonist and antagonist muscles themselves.

The concept of intermuscular synchronization plays a pivotal role in optimizing complex motor tasks such as those found in elite-level karate. This synchronization ensures that agonist, antagonist, and synergist muscles work in harmony [52], especially during rapid, explosive movements and during transitions between attack and defense. This coordination is highly dependent on both central nervous system (CNS) mechanisms and the integration of sensory feedback—particularly visual information [53]. A study by Danna-Dos-Santos et al. [54] emphasized the significance of visual input on the coordination of postural muscles. In karate, where maintaining a dynamic stance is crucial for both offensive and defensive maneuvers [32], this finding has important implications. The study [54] demonstrated that visual cues enhance low-frequency intermuscular coherence during quiet standing. This can be extrapolated to suggest that elite karate practitioners (KE), who must constantly adapt to the opponent’s position and movement, benefit from heightened visual-motor integration that enhances stability and readiness. Further, the work of Nguyen et al. [53] sheds light on the relative contribution of different sensory modalities. Their finding that somatosensory input plays a more dominant role than visual input in modulating intermuscular coherence during bimanual tasks suggests that in high-level karate, where tactile feedback (e.g., from blocking or parrying) is rich and immediate, somatosensory systems may become increasingly prioritized. This may explain the more refined neuromuscular control observed in KE and KSE groups, as repeated sparring likely enhances proprioceptive acuity [55].

In terms of movement execution, Zipser-Mohammadzada et al. [56] demonstrated that visually guided walking increases intramuscular coherence at higher frequencies (15–55 Hz), indicating greater supraspinal involvement. This aligns well with karate biomechanics, where rapid footwork and direction changes are often visually cued. During kumite, the need to constantly adapt to the opponent’s motion likely recruits high-frequency neural synchronization, particularly in muscles responsible for balance and propulsion. Kenville et al. [57] focused on the modulation of intermuscular coherence during the phases of bipedal squatting, showing distinct neural control strategies across eccentric and concentric movement phases. This is particularly relevant for karate techniques such as “mae geri” (front kick) or “gyaku zuki” (reverse punch), where lower limb movements alternate between loading and execution phases [47]. The ability to maintain coherence during both dynamic and static phases may distinguish elite performers from less experienced athletes. Lastly, de Vries et al. [58] provided evidence that increased coordination demands during bimanual tasks result in higher intermuscular coherence and reduced corticomuscular coherence. This suggests a shift toward subcortical control as motor skill becomes more automatic—a hallmark of expert karate performance. Elite karateka likely display more efficient intermuscular synchronization, with less reliance on cortical oversight, allowing for faster and more reflexive responses during combat [14].

Taken together, these studies support the notion that elite karate athletes possess more refined intermuscular synchronization mechanisms, facilitated by enhanced visual and somatosensory integration and a greater reliance on subcortical motor control. The inclusion criteria of the KE and KSE groups (i.e., black belt status, national-level competition, medal performance) are indicative of long-term, high-quality motor learning that likely fosters these adaptations. In contrast, the NA and KB groups, with minimal or no karate training, are less likely to exhibit such efficient neuromuscular coordination. These distinctions should be further explored in future studies using intermuscular coherence measures to assess training-induced neuroplasticity across levels of karate expertise.

A key strength of this study is its diverse sample, which offers a new perspective on the contractile properties of skeletal muscles in general, as well as among karate practitioners with varying levels of training. One of the main advantages of the TMG testing method, compared to stress-based assessments of contractile characteristics, is that it does not induce fatigue. As such, it does not disrupt training planning or periodization, allowing for more frequent testing. Additionally, this method minimizes the influence of external factors such as motivation or attentional fluctuations. Given that the coordination indexes derived from field-based TMG measurements effectively detected mechanical differences across karate subgroups, this approach can be reliably used by coaches and sports scientists to safely and efficiently assess athletes’ muscle function.

Some considerations should be taken into account when interpreting the results of this study. First of all, only individuals of one gender were analyzed; therefore, in future research, the neuromuscular characteristics of karate subgroups in the female population should be examined. Secondly, we recommend studying more muscles related to the movement and/or execution of kicks—for example the gastrocnemius muscle. As a final remark, the number of subjects in the KE group was relatively small, due to the high standards for inclusion.

## 5. Conclusions

In the present article, we investigated whether adjustments in neuromuscular coordination may emerge with karate training. More precisely, we examined the intermuscular synchronization of the knee extensor and flexor muscles and its level-dependency in karate athletes using a coherence analysis between TMG indexes. Our findings confirm this relationship, enhancing our understanding of neuromuscular adaptations among elites, sub-elites, basically trained karateka, and non-athletes, which could potentially contribute to advancing the overall quality of the sport. Indexes of Intermuscular Synchronization (IIS) offer a novel perspective for understanding the neuromechanical contractile properties of muscles. Differences in time (Tc and TcT) and speed (RMTD) IIS of the knee joint flexors and extensors of the dominant and non-dominant legs between the observed groups indicate the existence of the functional and hierarchical structure homogenization of neuromechanical strategies, which is positively related to competitive success and level of training. The established number of IIS differences suggests highly refined coordination in the knee flexor and extensor synergist muscles in elite karate athletes.

## Figures and Tables

**Table 1:** Descriptive characteristics of the study participants.

		Age	BH (cm)	BM (kg)	BMI (kg/m²)
Elite karate athletes (*n* = 7)	Average	28.67	184.63	86.43	25.38
	SD	2.66	2.76	7.02	2.38
	cV%	9.27	1.49	8.12	9.38
	Min	26.00	181.50	75.40	22.37
	Max	32.00	188.20	94.00	28.38
	Range	6.00	6.70	18.60	6.01
Sub-elite karate athletes (*n* = 14)	Average	21.79	180.56	77.12	23.67
	SD	3.12	9.07	6.89	1.61
	cV%	14.31	5.02	8.94	6.80
	Min	18.00	161.40	63.00	19.38
	Max	27.00	193.10	87.90	25.47
	Range	9.00	31.70	24.90	6.09
Basic karate group (*n* = 16)	Average	20.13	183.44	80.88	24.05
	SD	0.96	8.44	9.17	2.27
	cV%	4.76	4.60	11.34	9.45
	Min	19.00	168.50	69.30	18.40
	Max	23.00	197.10	98.00	26.77
	Range	4.00	28.60	28.70	8.36
Non–athletes (*n* = 14)	Average	26.07	180.70	82.77	25.28
	SD	3.75	7.23	13.30	3.44
	cV%	14.39	4.00	16.07	13.61
	Min	19.00	170.50	60.80	19.30
	Max	30.00	196.00	106.30	31.80
	Range	11.00	25.50	45.50	12.50

Note: BH—body height; BM—body mass; BMI—body mass index; SD—standard deviation; cV%—percent coefficient of variation; Min—minimum; Max—maximum.

**Table 2:** Results of the Indexes of Intermuscular Synchronization (IIS) of the dominant extensor and flexor muscles in the knee joint for the contraction time (Tc) parameter.

	Groups				ANOVA			
	Karate Elite	Karate Sub-Elite	Karate Basic	Non-Athletes	F	Sig.	Part. Eta^2^	Power
**IIS_AVG_Tc_Q_D**	25.14 ± 2.07 *	25.34 ± 2.07 **^§^**	24.97 ± 3.64 **^Ø^**	28.69 ± 3.71	4.400	**0.008**	0.219	0.845
**IIS** **_cV_Tc_Q_D**	9.13 ± 5.11 *	15.95 ± 8.32	17.29 ± 6.50	20.76 ± 12.53	2.807	**0.050**	0.149	0.624
**IIS_AVG_Tc_H_D**	31.35 ± 7.58	39.03 ± 7.48	35.70 ± 8.45	35.45 ± 8.97	1.400	0.254	0.082	0.348
**IIS** **_cV___Tc_H_D**	36.38 ± 20.48	25.93 ± 14.21	23.48 ± 14.35	22.55 ± 18.13	1.274	0.294	0.075	0.318

Note: IIS—Index of Intermuscular Synchronization; AVG—average; cV—coefficient of variation; Q—muscles extensors of the knee joint; H—muscles flexors of the knee joint; D—dominant leg. Statistical significances between the groups were marked as follows: KE and NA—*; KSE and NA—§; KB and NA—Ø. Parameters are expressed in milliseconds.

**Table 3:** Results of the Indexes of Intermuscular Synchronization (IIS) of the non-dominant extensors and flexors muscles in the knee joint for the parameter contraction time (Tc).

	Groups				ANOVA			
	Karate Elite	Karate Sub-Elite	Karate Basic	Non-Athletes	F	Sig.	Part. Eta^2^	Power
**IIS_AVG_Tc_Q_ND**	23.81 ± 1.74	24.48 ± 3.18 **^§^**	24.90 ± 5.42	28.62 ± 3.00	3.889	**0.015**	0.199	0.793
**IIS** **_cV_Tc_Q_ND**	10.07 ± 4.59	14.55 ± 6.54	17.43 ± 9.16	18.45 ± 10.97	1.752	0.169	0.101	0.428
**IIS_AVG_Tc_H_ND**	27.87 ± 5.96 ^¥@^*	42.15 ± 9.29	38.05 ± 8.47	41.78 ± 5.50	6.298	**0.001**	0.287	0.952
**IIS** **_cV_Tc_H_ND**	22.92 ± 15.96	23.40 ± 22.74	28.88 ± 17.27	24.83 ± 17.69	0.277	0.842	0.017	0.099

Note: IIS—Index of Intermuscular Synchronization; AVG—average; cV—coefficient of variation; Q—muscles extensors of the knee joint; H—muscles flexors of the knee joint; ND—non-dominant leg. Statistical significance between the groups were marked as follows: KE and KSE—¥; KE and KB—@; KE and NA—*; KSE and NA—§. Parameters are expressed in milliseconds.

**Table 4:** Results of the Indexes of Intermuscular Synchronization (IIS) of the dominant extensors and flexors muscles in the knee joint for the parameter total contraction time (TcT).

	Groups				ANOVA			
	Karate Elite	Karate Sub-Elite	Karate Basic	Non-Athletes	F	Sig.	Part. Eta^2^	Power
**IIS_AVG_TcT_Q_D**	48.37 ± 3.20	47.40 ± 2.76 **^§^**	47.03 ± 4.62 **^Ø^**	51.92 ± 4.36	4.613	**0.007**	0.227	0.863
**IIS** **_cV_TcT_Q_D**	10.38 ± 8.47	11.36 ± 6.05	11.67 ± 4.74	13.45 ± 7.93	0.417	0.741	0.026	0.127
**IIS_AVG_TcT_H_D**	53.85 ± 8.69	62.79 ± 8.41	58.92 ± 10.29	61.30 ± 11.47	1.393	0.256	0.082	0.346
**IIS** **_cV_TcT_H_D**	23.65 ± 15.03	18.94 ± 9.85	16.28 ± 9.81	17.10 ± 3.58	0.706	0.553	0.043	0.188

Note: IIS—Index of Intermuscular Synchronization; AVG—average; cV—coefficient of variation; Q—muscles extensors of the knee joint; H—muscles flexors of the knee joint; D—dominant leg. Statistical significance between the groups were marked as follows: KE and KSE and NA—§; KB and NA—Ø. Parameters are expressed in milliseconds.

**Table 5:** Results of the Indexes of Intermuscular Synchronization (IIS) of the non-dominant extensors and flexors muscles in the knee joint for the parameter total contraction time (TcT).

	Groups				ANOVA			
	Karate Elite	Karate Sub-Elite	Karate Basic	Non-Athletes	F	Sig.	Part. Eta^2^	Power
**IIS_AVG_TcT_Q_ND**	45.07 ± 2.77 *	45.68 ± 4.50 **^§^**	46.41 ± 6.67 **^Ø^**	52.20 ± 4.03	5.474	**0.003**	0.259	0.919
**IIS** **_cV___TcT_Q_ND**	5.91 ± 2.60	9.54 ± 4.98	11.56 ± 6.42	13.18 ± 6.83	2.740	0.054	0.149	0.627
**IIS_AVG_TcT_H_ND**	49.41 ± 6.71 ^¥@^*	65.64 ± 10.84	61.19 ± 9.29	65.82 ± 7.86	6.070	**0.001**	0.279	0.945
**IIS** **_cV_TcT_H_ND**	14.65 ± 9.22	17.01 ± 16.32	19.91 ± 11.22	19.87 ± 15.03	0.341	0.795	0.021	0.112

Note: IIS—Index of Intermuscular Synchronization; AVG—average; cV—coefficient of variation; Q—muscles extensors of the knee joint; H—muscles flexors of the knee joint; ND—non-dominant leg. Statistical significance between the groups were marked as follows: KE and KSE—¥; KE and KB—@; KE and NA—*; KSE and NA—§; KB and NA—Ø. Parameters are expressed in milliseconds.

**Table 6:** Results of the Indexes of Intermuscular Synchronization (IIS) of the dominant extensors and flexors muscles in the knee joint for the parameter rate of muscle tension development (RMTD).

	Groups				ANOVA			
	Karate Elite	Karate Sub-Elite	Karate Basic	Non-Athletes	F	Sig.	Part. Eta^2^	Power
**IIS_AVG_RMTD_Q_D**	0.27 ± 0.06	0.25 ± 0.05	0.24 ± 0.09	0.21 ± 0.07	1.576	0.208	0.091	0.388
**IIS** **_cV_RMTD_Q_D**	16.20 ± 10.74 **^¥@^**	34.85 ± 11.94	43.20 ± 16.36	32.80 ± 14.34	6.105	**0.001**	0.280	0.946
**IIS_AVG_RMTD_H_D**	0.21 ± 0.04 *	0.20 ± 0.04 **^§^**	0.18 ± 0.07	0.13 ± 0.04	5.350	**0.003**	0.255	0.912
**IIS** **_cV_RMTD_H_D**	18.98 ± 12.96	16.57 ± 7.86	21.32 ± 14.14	19.46 ± 16.68	0.316	0.814	0.020	0.107

Note: IIS—Index of Intermuscular Synchronization; AVG—average; cV—coefficient of variation; Q—muscles extensors of the knee joint; H—muscles flexors of the knee joint; D—dominant leg. Statistical significance between the groups were marked as follows: KE and KSE—¥; KE and KB—@; KE and NA—*; KSE and NA—§. Parameters are expressed in millimeters/milliseconds.

**Table 7:** Results of the Indexes of Intermuscular Synchronization (IIS) of the non-dominant extensors and flexors muscles in the knee joint for the parameter rate of muscle tension development (RMTD).

	Groups				ANOVA			
	Karate Elite	Karate Sub-Elite	Karate Basic	Non-Athletes	F	Sig.	Part. Eta^2^	Power
**IIS_AVG_RMTD_Q_ND**	0.29 ± 0.05 ^@^*	0.21 ± 0.06	0.19 ± 0.08	0.20 ± 0.06	3.980	**0.013**	0.203	0.803
**IIS** **_cV_RMTD_Q_ND**	16.32 ± 5.87	22.66 ± 14.73	27.94 ± 22.86	25.97 ± 21.30	0.686	0.565	0.042	0.184
**IIS_AVG_RMTD_H_ND**	0.22 ± 0.04 *	0.19 ± 0.04	0.18 ± 0.07	0.14 ± 0.06	3.713	**0.018**	0.192	0.772
**IIS** **_cV_RMTD_H_ND**	18.28 ± 14.16	21.37 ± 13.92	26.49 ± 17.23	18.53 ± 16.79	0.783	0.510	0.048	0.205

Note: IIS—Index of Intermuscular Synchronization; AVG—average; cV—coefficient of variation; Q—muscles extensors of the knee joint; H—muscles flexors of the knee joint; ND—non-dominant leg. Statistical significance between the groups were marked as follows: KE and KB—@; KE and NA—*. Parameters are expressed in millimeters/milliseconds.

## Data Availability

The data presented in this study are available on request from the corresponding author as requested by coaches of participating athletes.
